# miR-29a-3p directly targets Smad nuclear interacting protein 1 and inhibits the migration and proliferation of cervical cancer HeLa cells

**DOI:** 10.7717/peerj.10148

**Published:** 2020-10-20

**Authors:** Ying Chen, Weiji Zhang, Lijun Yan, Peng Zheng, Jin Li

**Affiliations:** 1 College of Life Science, Yangtze University, Jingzhou, Hubei, China; 2 Institute of Biology and Medicine, College of Life Science and Healthy, Wuhan University of Science and Technology, Wuhan, Hubei, China

**Keywords:** SNIP1, miR-29a-3p, Migration, Proliferation, Cervical cancer

## Abstract

Smad nuclear interacting protein 1 (SNIP1) is a nuclear protein and involved in essential biological processes. MicroRNAs are effective regulators of tumorigenesis and cancer progression via targeting multiple genes. In present study, we aimed to investigate the function of SNIP1 and identify novel miRNA-SNIP1 axis in the development of cervical cancer. The results showed for the first time that silencing of the *SNIP1* gene inhibited the migration and proliferation in HeLa cells significantly. Bioinformatics analysis and dual luciferase reporter assay demonstrated that miR-29a-3p could target 3′ UTR of SNIP1 directly. The mRNA and protein expression levels of SNIP1 were negative regulated by miR-29a-3p according to the RT-qPCR and Western blot analysis, respectively. Furthermore, functional studies showed that over-expression of miR-29a-3p restrained HeLa cells migration and proliferation, and the mRNA expression of SNIP1 downstream genes (*HSP27*, *c-Myc*, and *cyclin D1*) were down-regulated by miR-29a-3p. Together, we concluded that miR-29a-3p suppressed the migration and proliferation in HeLa cells by directly targeting SNIP1. The newly identified miR-29a-3p/SNIP1 axis could provide new insight into the development of cervical cancer.

## Introduction

Cervical cancer is the fourth most prevalent malignancy and remains the major cause of cancer death among females worldwide (Bray et al., 2018). Despite at there are considerable improvements of treatment strategies in the therapy of cervical cancer, the overall 5-year survival rates of patients remain poor for metastasis (Forouzanfar et al., 2011; Tewari et al., 2014; Li, Wu & Cheng, 2016). Therefore, understanding the pathogenesis and progression of cervical cancer is quite necessary and may facilitate identification of effective therapeutic targets for cervical cancer treatment.

Smad nuclear interacting protein 1 (SNIP1), as an evolutionarily conserved nuclear protein, is involved in essential biological processes such as cell proliferation (Roche et al., 2004; Fujii et al., 2006), small RNA biogenesis (Yu et al., 2008), DNA damage response (Chen et al., 2018), and several signaling pathways (Kim et al., 2000; Kim et al., 2001). In patients with non-small cell lung cancer or tongue squamous cell carcinoma, SNIP1 might be a reliable prognostic indicator (Liang et al., 2011; Jeon et al., 2013). Recently, SNIP1 was considered to be targeted by microRNA-335 and involved in osteosarcoma proliferation and metastasis (Xie et al., 2019). However, the exact role of SNIP1 in the development of cervical cancer remains obscure.

MicroRNAs (miRNAs) are a group of non-coding RNAs with 19-25 nucleotides and have closely relationships with the occurrence and progression of human cancers (Romano et al., 2017). MicroRNAs regulate gene expression mainly through binding to the 3′-untranslated region (UTR) of target mRNAs (Bartel, 2004). As oncogene or tumor suppressor in different cancers, miRNAs play essential roles in basic biological processes such as cell proliferation, apoptosis, differentiation, migration and invasion (Bartel, 2004; Pardini et al., 2018). Accumulating studies have shown that miR-29a was abnormally expressed in various cancers, including cervical cancer (Pei, Lei & Liu, 2016; Yang et al., 2017; Gong et al., 2019). However, the detailed role and underlying mechanism of miR-29a in cervical cancer is still largely unclear.

In this research, we investigated the biological role of SNIP1 in the progression of cervical cancer. Furthermore, we predicted and demonstrated that miR-29a-3p inhibited the transcription and protein expression of SNIP1 by targeting 3′ UTR directly, and suppressed the proliferation and migration of cervical cancer cells.

## Materials & Methods

### Cell culture

The human cervical cancer HeLa cells were purchased from American Type Culture Collection (Manassas, VA), and maintained in Dulbecco’s modified Eagle’s medium (Hyclone, USA) containing 10% fetal bovine serum (Gibco, USA) under a humidified environment with 5% CO_2_ at 37 °C.

### Transfection

Three small interfering RNAs (siRNAs) targeting SNIP1 (siSNIP1-330, siSNIP1-871, siSNIP1-1059) were purchased from GenePharma (Suzhou, China). The miR-29a-3p mimics and negative control (NC) were manufactured by RiboBio (Guangzhou, China). All transfections were performed using siRNA-Mate reagent (GenePharma, China) in accordance with the instruction manual. The cells were collected after 48 h of transfection for further experiments.

### Quantitative RT-PCR (RT-qPCR)

Total RNA was harvested from HeLa cells by EZNA Total RNA Kit (Omega BioTek, USA). The first-strand cDNA was generated using HiScript II Q RT SuperMix (Vazyme, China). RT-qPCR was performed to quantify relative RNA levels using ChamQ Universal SYBR qPCR Master Mix (Vazyme, China) on a CFX96 Touch (Bio-rad, USA). The 2^−ΔΔ*Ct*^ method was used to measure the relative expression level, and GAPDH served as the internal reference. The primers used for RT-qPCR were presented in Table 1.

**Table 1:** Primers for quantitative RT-PCR.

**Gene**	**Forward 5′–3′**	**Reverse 5′–3′**
*SNIP1*	GCTTTGTGGACCAGGTGTTT	TGTACAGTCACGGGCTTGAG
*cyclin D1*	TTTGTTGTGTGTGCAGGGAG	TTTCTTCTTGACTGGCACGC
*CDK2*	TGAAGATGGACGGAGCTTGT	ACTGGAGGAGAGGGTGAGAT
*MMP9*	GCGTCTTCCCCTTCACTTTC	ATAGGGTACATGAGCGCCTC
*MAPK1*	GAACTTCTGCAACCCCACTG	CAGCCGCAGTTATAAGCAGG
*N-cadherin*	GACAATGCCCCTCAAGTGTT	CCATTAAGCCGAGTGATGGT
*E-cadherin*	CGGACGATGATGTGAACACC	TTGCTGTTGTGCTTAACCCC
*HSP27*	AGTGGTCGCAGTGGTTAGG	TCCTTGGTCTTGACCGTCAG
*c-Myc*	AACACACAACGTCTTGGAGC	GCACAAGAGTTCCGTAGCTG
*VIM*	AGCTAACCAACGACAAAGCC	TTGCGTTCAAGGTCAAGACG
*GAPDH*	CGACCACTTTGTCAAGCTCA	AGGGGTCTACATGGCAACTG

### Western blot analysis

The cultured cells were lysed with RIPA buffer (Beyotime, China). The protein concentration was quantified by Enhanced BCA Assay kit (Beyotime, China). Total protein samples were separated with 10% SDS-PAGE gel and transferred onto PVDF membrane (Millipore, USA). After blocked with 5% skim milk, the membrane was probed with primary antibodies against SNIP1 (1:1,000, Proteintech, USA) and GAPDH (1:20,000, Proteintech, USA) at 4 °C overnight and followed by incubation with secondary antibodies (1:4,000, Beyotime, China). Blots were visualized by BeyoECL Plus Kit (Beyotime, China) and scanned with a ChemiDoc XRS imaging system (Bio-Rad, USA).

### Dual luciferase reporter assay

To determine the binding affinity between SNIP1 and miR-29a-3p, the recombinant psiCHECK-2 vectors (Promega, USA) with the wild type (WT) or mutant of *SNIP1* gene 3′-UTR were constructed. Then, the recombinant vectors (WT or mutant) and miR-29a-3p mimics (or NC) were co-transfected in HeLa cells. Dual-Luciferase Reporter Assay system (Promega, USA) was used to calculate the luciferase activity after transfection according to the manual instruction.

### Scratch assay

Transfected HeLa cells were cultured in 6-well plates until the confluence reached 100%. Then, a sterile pipet tip was used to scrape on the bottom of culture plates. Cell migration was observed at 12 h under an inverted microscope (Olympus, Japan) and images were captured for each sample. The scratch area was measured and analyzed by ImageJ software (NIH, MD, USA).

### Cell Counting Kit-8 assay

Hela cells (3,000 cells/well) were seeded into 96-well plates after transfection, and cultured for 24 h, 48 h, 72 h and 96 h. Fresh medium with 10% CCK-8 (Genview Scientific, AUS) was mixed carefully, and the absorbance values of 450 nm wavelength were detected at least three times by a spectrophotometric plate reader (Hitachi, Japan).

### Transwell migration assay

Transfected cells (5 × 10^4^) were placed into the top chamber of the transwell inserts (Corning, USA) and maintained with serum-free medium. Then the lower chamber was filled with complete medium. And the migrated cells were fixed and stained 24 h later. Finally, the cells stained in more than three visual fields were randomly selected and photographed with an inverted microscope (Olympus, Japan) and counted.

### Statistical analysis

Statistical analysis was carried out using the GraphPad Prism 8 (GraphPad Software, SanDiego, USA) program. All results were presented as mean ± standard error (SD) of three independent experiments. Comparison among multiple groups was assessed by Student’s *t* test. A statistically significant difference was defined as *P* < 0.05.

## Results

### Knockdown of SNIP1 reduced migration and proliferation in cervical cancer cells

**Figure 1:**
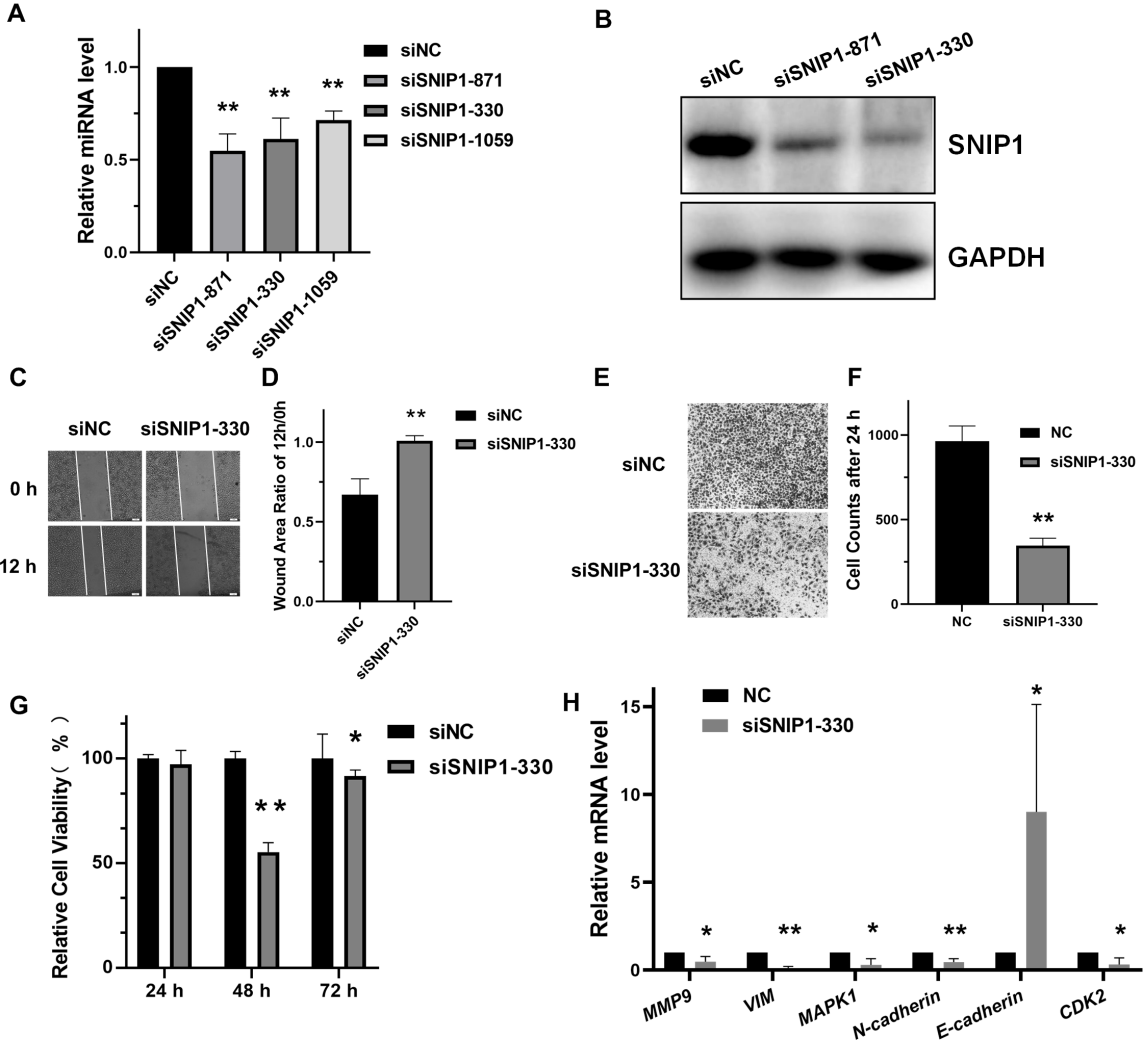
Knockdown of SNIP1 inhibited HeLa cells migration and proliferation. (A–B) RT-qPCR and Western blot analysis of SNIP1 expression in different siRNAs transfected HeLa cells. (C–F) Scratch assay and transwell analysis were performed to determine the migration of HeLa cells transfected with siSNIP1-330 or siNC, respectively. (G) Cell viability was measured using the CCK-8 assay after knockdown SNIP1 in HeLa cells at 24 h, 48 h, and 72 h. (H) RT-qPCR analysis of migration-related genes and proliferation-related genes in HeLa cells infected with siSNIP1-330 or siNC, respectively. GAPDH was used as internal control. Each experiment were repeated three times, *P < 0.05, **P < 0.01.

To address the biological role of SNIP1 in the progression of cervical cancer, three small interference RNAs (siRNAs) targeting SNIP1 were synthesized and transfected into HeLa cells. Among them, siSNIP1-330 showed the best silencing effect (Figs. 1A and 1B), which was subsequently chosen for further analysis. After transfected with siSNIP1-330, the wound area ratio increased (Figs. 1C and 1D) and the number of migrated cells decreased (Figs. 1E and 1F), which demonstrated the migration of HeLa cells was suppressed. Furthermore, the cell proliferation rate declined after transfection for 48 h (Fig. 1G). In addition, the mRNA expression levels of several migration-related genes (*MMP9*, *VIM*, *MAPK1*, *N-cadherin* and *E-cadherin*) and proliferation-related genes (*CDK2*) in HeLa cells (Wang & Chen, 2019) can also be downregulated or upregulated (Fig. 1H). Hence, SNIP1 knockdown could reduce migration and proliferation in cervical cancer HeLa cells.

### SNIP1 was directly targeted by miR-29a-3p

**Figure 2:**
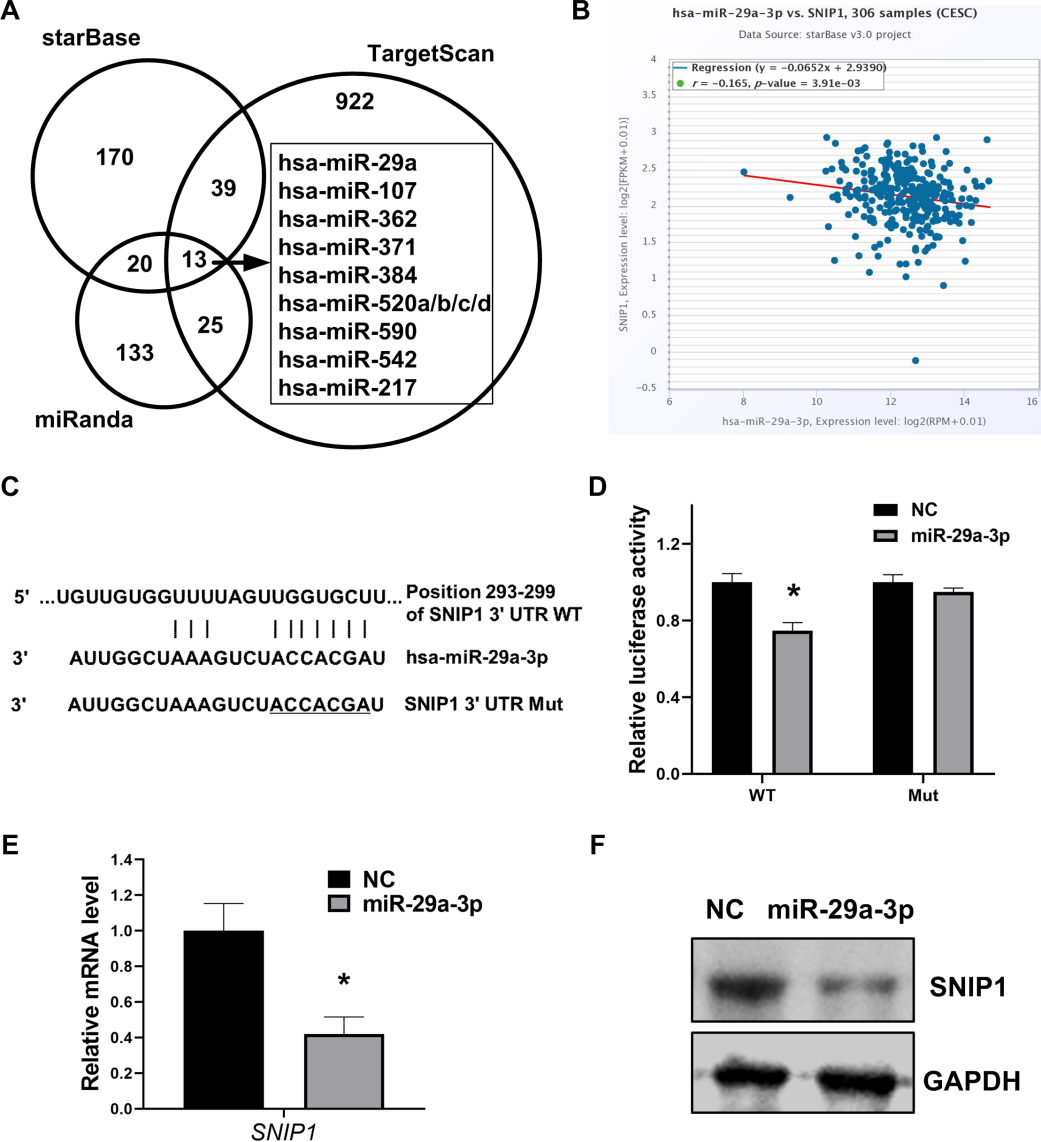
miR-29a-3p directly targets SNIP1 in HeLa cells. (A) Potential miRNAs binding SNIP1 predicted in starBase, TargetScan and miRanda. (B) Correlation between miR-29a-3p with SNIP1 in CESC samples. (C) Graphical presentation of putative binding sites for miR-29a-3p in the wild type and mutant type 3′ UTR of SNIP1. (D) Dual-luciferase reporter assay was performed following transfection with miR-29a-3p mimics or NC mimics using psi-check2 luciferase vector including the SNIP1 WT 3′ UTR or the mutant 3′ UTR. (E) After transfection of miR-29a-3p mimics or NC mimics in HeLa cells, SNIP1 mRNA expression were detected by RT-qPCR. (F) SNIP1 protein expression was measured by Western blotting after transfection. GAPDH served as the internal control. *P < 0.05.

To further confirm the exact miRNA that can directly target SNIP1, three different bioinformation tools starBase, TargetScan and miRanda, were performed to calculate the possibility scores. Intersection of these three sets showed that there were 12 candidate miRNAs which target SNIP1(Fig. 2A), and miR-29a-3p was chosen for the highest score (Table 2). Moreover, the expression relationship between miR-29a-3p and SNIP1 is negative correlation (*P* < 0.05) in cervical squamous cell carcinoma and endocervical adenocarcinoma (CESC) samples from starBase (Fig. 2B, Table 2). Compared with the control group, the luciferase activity was inhibited significantly in HeLa cells when co-transfected with SNIP1 wild type 3′-UTR vector and miR-29a-3p mimics (Figs. 2C and 2D). Furthermore, both mRNA and protein expression levels of SNIP1 in HeLa cells were declined after transfection with miR-29a-3p mimics (Figs. 2E and 2F). Taken together, these results demonstrated that miR-29a-3p targeted SNIP1 via directly binding its 3′ UTR region and negatively regulated SNIP1 expression in cervical cancer.

**Table 2:** Potential miRNAs binding SNIP1 and co-expression analysis for the miRNA-SNIP1 in CESC.

miRNA	Position in the UTR^a^	Seed match^a^	Context++ score percentile^a^	*r*^b^	*p*-value^b^
hsa-miR-29a-3p	293–299	7mer-m8	96	−0.165	3.91E−03
hsa-miR-542-3p	836–842	7mer-m8	94	−0.057	3.19E−01
hsa-miR-384	314–321	8mer	91	0.000	1.00E +00
hsa-miR-520d-5p	361–367	7mer-m8	90	0.023	6.91E−01
hsa-miR-371a-5p	504–510	7mer-m8	89	−0.034	5.59E−01
hsa-miR-520a-3p	535–541	7mer-1A	88	0.098	8.66E−02
hsa-miR-520b	535–541	7mer-1A	87	0.012	8.29E−01
hsa-miR-520c-3p	535–541	7mer-1A	87	0.017	7.64E−01
hsa-miR-362-3p	1,058–1,064	7mer-1A	66	−0.034	5.59E−01
hsa-miR-590-3p	1,191–1,197	7mer-m8	59	−0.149	8.83E−03
hsa-miR-107	2,140–2,146	7mer-1A	46	0.013	8.15E−01
hsa-miR-217	2,143–2,149	7mer-1A	46	−0.083	1.50E−01

**Notes.**

CESC, cervical squamous cell carcinoma and endocervical adenocarcinoma.

a Analysis from TargetScan7.2 (http://www.targetscan.org/vert_72/).

b Analysis from starBase v3.0 pan-cancer analysis (http://starbase.sysu.edu.cn/panMirCoExp.php).

### MiR-29a-3p inhibited migration and proliferation in cervical cancer cells

**Figure 3:**
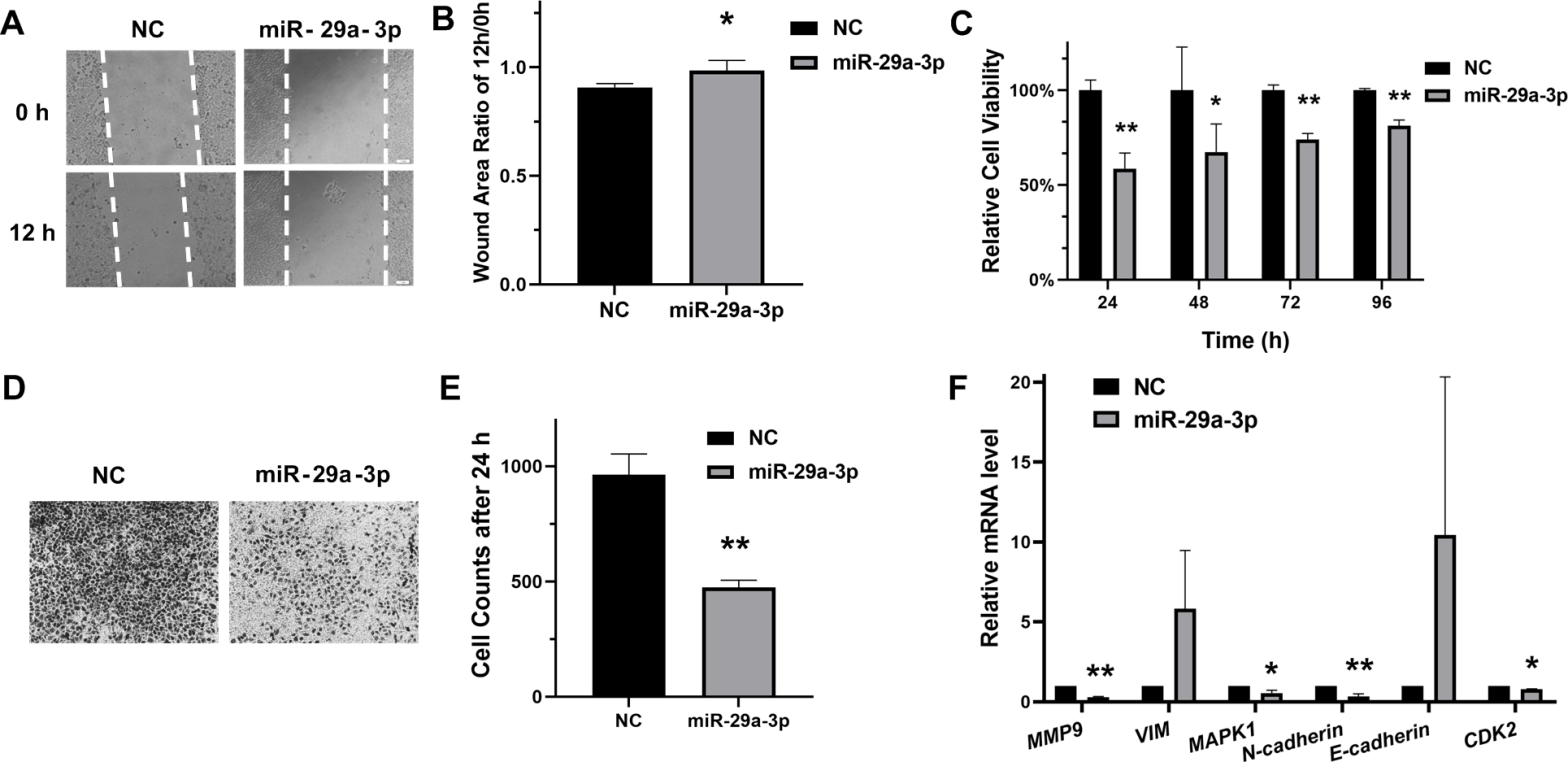
MiR-29a-3p inhibited HeLa cells migration and proliferation. (A–B) Cell scratch assay to evaluate HeLa cells migration after transfection of miR-29a-3p mimics or NC, respectively. The ratio of area 12 h/0 h was measured by Image J. (C) Cell viability was measured after transfection of miR-29a-3p mimics or NC. (D–E) The effects of miR-29a-3p on migration were determined by transwell assay in HeLa cells. (F) RT-qPCR analysis of migration-related genes and proliferation-related genes in HeLa cells transfected with miR-29a-3p mimics or NC. *P < 0.05, **P < 0.01.

To evaluate the regulatory roles of miR-29a-3p in HeLa cells, the scratch assay and transwell assay for migration were performed. As results shown in Figs. 3A, 3B, 3D and 3E, transfection with miR-29a-3p mimics restrained migration in HeLa cells. Moreover, miR-29a-3p mimics also significantly decreased the relative cell viability in HeLa cells (Fig. 3C). Additionally, miR-29a-3p also regulated the mRNA expression levels of genes associated with migration or proliferation in HeLa cells (Fig. 3F). Therefore, these data indicated that miR-29a-3p inhibited migration and proliferation in cervical cancer cells.

### MiR-29a-3p regulated the mRNA expression of SNIP1 downstream genes

**Figure 4:**
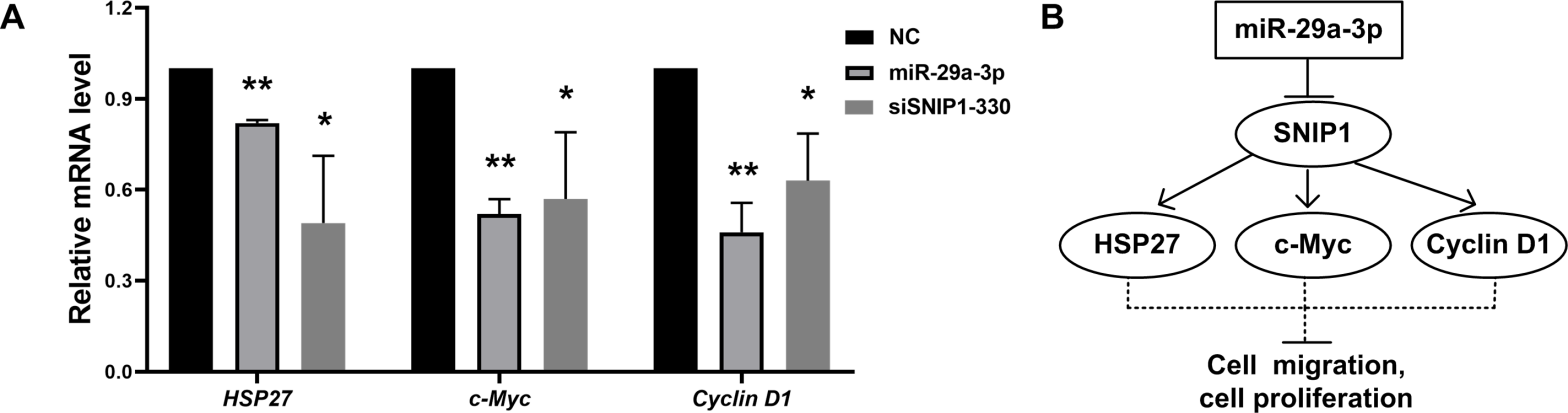
MiR-29a-3p regulated the downstream genes of SNIP1. (A) The relative mRNA level of downstream genes of SNIP1 (*HSP27*, *c-Myc* and *cyclin D1*) in HeLa cells transfected with miR-29a-3p mimics, siSNIP1-330 or NC were determined by RT-qPCR. (B) Schematic diagram of miR-29a-3p and SNIP1 regulate migration and proliferation in HeLa cells. *P < 0.05, **P < 0.01.

To investigate whether miR-29a-3p would have effects on the downstream of SNIP1, the mRNA levels of these downstream genes (*HSP27*, *c-Myc* and C*yclin D1*) (Fujii et al., 2006; Bracken et al., 2008; Zhu et al., 2010) were detected by RT-qPCR after transfection with miR-29a-3p mimics or siSNIP1-330. The data suggested that miR-29a-3p and knockdown of SNIP1 both reduced the expression of those genes significantly (Fig. 4A).

## Discussion

SNIP1 is a transcription regulator contains a nuclear localization sequence, and plays a key role in tumor development and progression (Kim et al., 2000; Kim et al., 2001; Fujii et al., 2006; Bracken et al., 2008). Over-expression of SNIP1 promoted cell invasion and migration in osteosarcoma cells (Xie et al., 2019). And the knockdown of SNIP1 restrained the anchorage-independent growth of lung cancer cells (Jeon et al., 2013). However, the function of SNIP1 in cervical cancer development is poorly understood. In our analysis, the migration and proliferation of cervical cancer cells was significantly suppressed after siRNA-mediated silencing of SNIP1 (Figs. 1C, 1D, 1E, 1F and 1G). These results suggested that SNIP1 was involved in the development of HeLa cells as an oncogene.

As a member of the miR-29 family, miR-29a is considered to play a crucial role in the regulation of multiple cancers (Wang et al., 2018). It was up-regulated and promoted epithelial-mesenchymal transition, migration and invasion in breast cancer cells (Wu et al., 2019). In contrast, other studies have shown that miR-29a was down-regulated and inhibited the progression of cancers such as colon cancer (Shi et al., 2020), non-small cell lung cancer (Hu et al., 2016) and adenocarcinoma (Zhang et al., 2018). Previous studies have shown that miR-29a was low expression in cervical cancer, as a tumor suppressor, miR-29a can target multiple genes, such as CDC42, HSP47, SIRT1 and DNMT1 (Park et al., 2009; Yamamoto et al., 2013; Gong et al., 2019; Nan et al., 2019). Nonetheless, the detailed biological function of miR-29a in cervical cancer has not yet been completely revealed. In this research, we showed for the first time that SNIP1 was targeted by miR-29a-3p in cervical HeLa cells. The results indicated that miR-29a-3p could down-regulate SNIP1 expression levels (Figs. 2E and 2F), and the direct binding site of SNIP1 mRNA 3′ UTR was confirmed by dual luciferase reporter assay (Fig. 2D). Furthermore, our data also supported previous reports (Yamamoto et al., 2013), indicating that miR-29a-3p can suppress the migration and proliferation of HeLa cells (Figs. 3A, 3B, 3C, 3D and 3E). Sharing the same seed region in miR-29 family (miR-29a/b/c), we inferred that SNIP1 may also be targeted by other miR-29s.

It has been reported that SNIP1 could improve the transcriptional activity of c-Myc (Fujii et al., 2006), and regulate the stability of Cyclin D1 mRNA (Bracken et al., 2008). Otherwise, SNIP1 could down-regulate the transcription of HSP27 (Zhu et al., 2010). These downstream genes (c-Myc, Cyclin D1 and HSP27) regulated by SNIP1 are considered to be closely associated with cell proliferation and migration (Evan et al., 1994; Zhu et al., 2010; Li et al., 2012; Pestell, 2013). In this research, miR-29a-3p decreased the mRNA expression levels of *c-Myc*, *Cyclin D1* and *HSP27* in HeLa cells as an upstream regulator (Fig. 4A). Therefore, miR-29a-3p may mediate the regulation of cell proliferation and migration in cervical cancer cells via downstream genes of SNIP1 (Fig. 4B). Further study is required to illustrate the underlying mechanism of miR-29a-3p/SNIP1 pathway in cervical cancer oncogenesis.

In addition, this research has some defects in the following aspects. Although it was confirmed that miR-29a-3p can target SNIP1 to inhibit the migration and proliferation of HeLa cells, whether over-expression of SNIP1 could supplement the inhibitory effect of miR-29a-3p should be further observed. It was more convinced to detect the protein levels related to migration, proliferation as well as downstream of SNIP1, while the mRNA levels were reduced markedly by miR-29a-3p or SNIP1 siRNA in HeLa cells. In this study, we have used only one cell line to verify the function of miR-29a-3p/SNIP1, more cell lines and more in vitro and in vivo experiments are needed to be carried out. Finally, miR-590-3p is worth exploring as miR-29a-3p for the Pearson correlation analysis suggested that miR-590-3p was negatively correlated with SNIP1 in CESC tissues (*r* =  − 0.149, *P* = 0.00883) (Table 2).

## Conclusions

In conclusion, miR-29a-3p suppressed the migration and proliferation of cervical cancer cells by directly targeting SNIP1, and could also down-regulate the mRNA expression of SNIP1 downstream genes such as c-Myc, Cyclin D1 and HSP27. The newly identified miR-29a-3p/SNIP1 axis may provide new insights into the understanding of the progression of cervical cancer, and represent an effective treatment target for cervical cancer.

##  Supplemental Information

10.7717/peerj.10148/supp-1 Data S1 Raw data

10.7717/peerj.10148/supp-2 Figure S1 Expression of SNIP1 in HeLa cells after transfected with siSNIP1 at 72 h (A-B) RT-qPCR and Western blot analysis of SNIP1 expression in HeLa cells after transfected with siSNIP1-330 or siNC at 48 h and 72 h, respectively.

10.7717/peerj.10148/supp-3 Figure S2 Expression of SNIP1 and miR-29a-3p in cancer samples (A) Differential expression levels of SNIP1 from cervical cancer samples vs normal samples analyzed from GEPIA (http://gepia.cancer-pku.cn/). (B) Protein expression of SNIP1 in different cancer patients analyzed from HPA (https://www.proteinatlas.org/). 5/11 patients with cervical cancer show medium expression. (C) RNA expression of SNIP1 in different cell lines analyzed from HPA (https://www.proteinatlas.org/).

10.7717/peerj.10148/supp-4 Table S1 Co-expression analysis for the miR-29a-3p-SNIP1 in different cancer samples (starbase)

## Additional Information and Declarations

### Data Availability

The following information was supplied regarding data availability: The raw measurements are available in the Supplemental Files.

## References

[ref-1] Bartel DP (2004). MicroRNAs: genomics, biogenesis, mechanism, and function. Cell.

[ref-2] Bracken CP, Wall SJ, Barré B, Panov KI, Ajuh PM, Perkins ND (2008). Regulation of cyclin D1 RNA stability by SNIP1. Cancer Research.

[ref-3] Bray F, Ferlay J, Soerjomataram I, Siegel RL, Torre LA, Jemal A (2018). Global cancer statistics 2018: GLOBOCAN estimates of incidence and mortality worldwide for 36 cancers in 185 countries. CA: A Cancer Journal for Clinicians.

[ref-4] Chen LL, Lin HP, Zhou WJ, He CX, Zhang ZY, Cheng ZL, Song JB, Liu P, Chen XY, Xia YK, Chen XF, Sun RQ, Zhang JY, Sun YP, Song L, Liu BJ, Du RK, Ding C, Lan F, Huang SL, Zhou F, Liu S, Xiong Y, Ye D, Guan KL (2018). SNIP1 recruits TET2 to regulate c-MYC target genes and cellular DNA damage response. Cell Reports.

[ref-5] Evan G, Harrington E, Fanidi A, Land H, Amati B, Bennett M (1994). Integrated control of cell proliferation and cell death by the c-myc oncogene. Philosophical Transactions of the Royal Society of London. Series B, Biological Sciences.

[ref-6] Forouzanfar MH, Foreman KJ, Delossantos AM, Lozano R, Lopez AD, Murray CJ, Naghavi M (2011). Breast and cervical cancer in 187 countries between 1980 and 2010: a systematic analysis. Lancet.

[ref-7] Fujii M, Lyakh LA, Bracken CP, Fukuoka J, Hayakawa M, Tsukiyama T, Soll SJ, Harris M, Rocha S, Roche KC, Tominaga SI, Jen J, Perkins ND, Lechleider RJ, Roberts AB (2006). SNIP1 is a candidate modifier of the transcriptional activity of c-Myc on E box-dependent target genes. Molecular Cell.

[ref-8] Gong Y, Wan JH, Zou W, Lian GY, Qin JL, Wang QM (2019). MiR-29a inhibits invasion and metastasis of cervical cancer via modulating methylation of tumor suppressor SOCS1. Future Oncology.

[ref-9] Hu Z, Cui Y, Zhou Y, Zhou K, Qiao X, Li C, Wang S (2016). MicroRNA-29a plays a suppressive role in non-small cell lung cancer cells via targeting LASP1. OncoTargets and Therapy.

[ref-10] Jeon HS, Choi YY, Fukuoka J, Fujii M, Lyakh LA, Song SH, Travis WD, Park JY, Jen J (2013). High expression of SNIP1 correlates with poor prognosis in non-small cell lung cancer and SNIP1 interferes with the recruitment of HDAC1 to RB in vitro. Lung Cancer.

[ref-11] Kim RH, Flanders KC, Reffey SBirkey, Anderson LA, Duckett CS, Perkins ND, Roberts AB (2001). SNIP1 inhibits NF-kappa B signaling by competing for its binding to the C/H1 domain of CBP/p300 transcriptional co-activators. The Journal of Biological Chemistry.

[ref-12] Kim RH, Wang D, Tsang M, Martin J, Huff C, De Caestecker MP, Parks WT, Meng X, Lechleider RJ, Wang T, Roberts AB (2000). A novel smad nuclear interacting protein, SNIP1, suppresses p300-dependent TGF-beta signal transduction. Genes & Development.

[ref-13] Li Q, An J, Liu X, Zhang M, Ling Y, Wang C, Zhao J, Yu L (2012). SNIP1: a new activator of HSE signaling pathway. Molecular and Cellular Biochemistry.

[ref-14] Li H, Wu X, Cheng X (2016). Advances in diagnosis and treatment of metastatic cervical cancer. Journal of Gynecologic Oncology.

[ref-15] Liang X, Zheng M, Jiang J, Zhu G, Yang J, Tang Y (2011). Hypoxia-inducible factor-1 alpha, in association with TWIST2 and SNIP1, is a critical prognostic factor in patients with tongue squamous cell carcinoma. Oral Oncology.

[ref-16] Nan P, Niu Y, Wang X, Li Q (2019). MiR-29a function as tumor suppressor in cervical cancer by targeting SIRT1 and predict patient prognosis. OncoTargets and Therapy.

[ref-17] Pardini B, De Maria D, Francavilla A, Di Gaetano C, Ronco G, Naccarati A (2018). MicroRNAs as markers of progression in cervical cancer: a systematic review. BMC Cancer.

[ref-18] Park SY, Lee JH, Ha M, Nam JW, Kim VN (2009). miR-29 miRNAs activate p53 by targeting p85 alpha and CDC42. Nature Structural & Molecular Biology.

[ref-19] Pei YF, Lei Y, Liu XQ (2016). MiR-29a promotes cell proliferation and EMT in breast cancer by targeting ten eleven translocation 1. Biochimica et Biophysica Acta.

[ref-20] Pestell RG (2013). New roles of cyclin D1. The American Journal of Pathology.

[ref-21] Roche KC, Wiechens N, Owen-Hughes T, Perkins ND (2004). The FHA domain protein SNIP1 is a regulator of the cell cycle and cyclin D1 expression. Oncogene.

[ref-22] Romano G, Veneziano D, Acunzo M, Croce CM (2017). Small non-coding RNA and cancer. Carcinogenesis.

[ref-23] Shi X, Valizadeh A, Mir SM, Asemi Z, Karimian A, Majidina M, Safa A, Yosefi B (2020). miRNA-29a reverses P-glycoprotein-mediated drug resistance and inhibits proliferation via up-regulation of PTEN in colon cancer cells. European Journal of Pharmacology.

[ref-24] Tewari KS, Sill MW, Long 3rd HJ, Penson RT, Huang H, Ramondetta LM, Landrum LM, Oaknin A, Reid TJ, Leitao MM, Michael HE, Monk BJ (2014). Improved survival with bevacizumab in advanced cervical cancer. The New England Journal of Medicine.

[ref-25] Wang JY, Chen LJ (2019). The role of miRNAs in the invasion and metastasis of cervical cancer. Bioscience Reports.

[ref-26] Wang JY, Zhang Q, Wang DD, Yan W, Sha HH, Zhao JH, Yang SJ, Zhang HD, Hou JC, Xu HZ, He YJ, Hu JH, Zhong SL, Tang JH (2018). MiR-29a: a potential therapeutic target and promising biomarker in tumors. Bioscience Reports.

[ref-27] Wu Y, Shi W, Tang T, Wang Y, Yin X, Chen Y, Zhang Y, Xing Y, Shen Y, Xia T, Guo C, Pan Y, Jin L (2019). miR-29a contributes to breast cancer cells epithelial-mesenchymal transition, migration, and invasion via down-regulating histone H4K20 trimethylation through directly targeting SUV420H2. Cell Death & Disease.

[ref-28] Xie Y, Deng H, Wei R, Sun W, Qi Y, Yao S, Cai L, Wang Y, Deng Z (2019). Overexpression of miR-335 inhibits the migration and invasion of osteosarcoma by targeting SNIP1. International Journal of Biological Macromolecules.

[ref-29] Yamamoto N, Kinoshita T, Nohata N, Yoshino H, Itesako T, Fujimura L, Mitsuhashi A, Usui H, Enokida H, Nakagawa M, Shozu M, Seki N (2013). Tumor-suppressive microRNA-29a inhibits cancer cell migration and invasion via targeting HSP47 in cervical squamous cell carcinoma. International Journal of Oncology.

[ref-30] Yang YL, Wang FS, Li SC, Tiao MM, Huang YH (2017). MicroRNA-29a alleviates bile duct ligation exacerbation of hepatic fibrosis in mice through epigenetic control of methyltransferases. International Journal of Molecular Sciences.

[ref-31] Yu B, Bi L, Zheng B, Ji L, Chevalier D, Agarwal M, Ramachandran V, Li W, Lagrange T, Walker JC, Chen X (2008). The FHA domain proteins DAWDLE in Arabidopsis and SNIP1 in humans act in small RNA biogenesis. Proceedings of the National Academy of Sciences of the United States of America.

[ref-32] Zhang Y, He S, Mei R, Kang Y, Duan J, Wei R, Xiang C, Wu Y, Lu X, Cai Z, Xiong L (2018). miR-29a suppresses IL 13 induced cell invasion by inhibiting YY1 in the AKT pathway in lung adenocarcinoma A549 cells. Oncology Reports.

[ref-33] Zhu Z, Xu X, Yu Y, Graham M, Prince ME, Carey TE, Sun D (2010). Silencing heat shock protein 27 decreases metastatic behavior of human head and neck squamous cell cancer cells in vitro. Molecular Pharmaceutics.

