# Supplementary figures and images for: miR-29a-3p directly targets Smad nuclear interacting protein 1 and inhibits the migration and proliferation of cervical cancer HeLa cells
Source: PeerJ. 2020 Oct 20;8:e10148. doi: 10.7717/peerj.10148 (PMC7583608; doi:10.7717/peerj.10148)

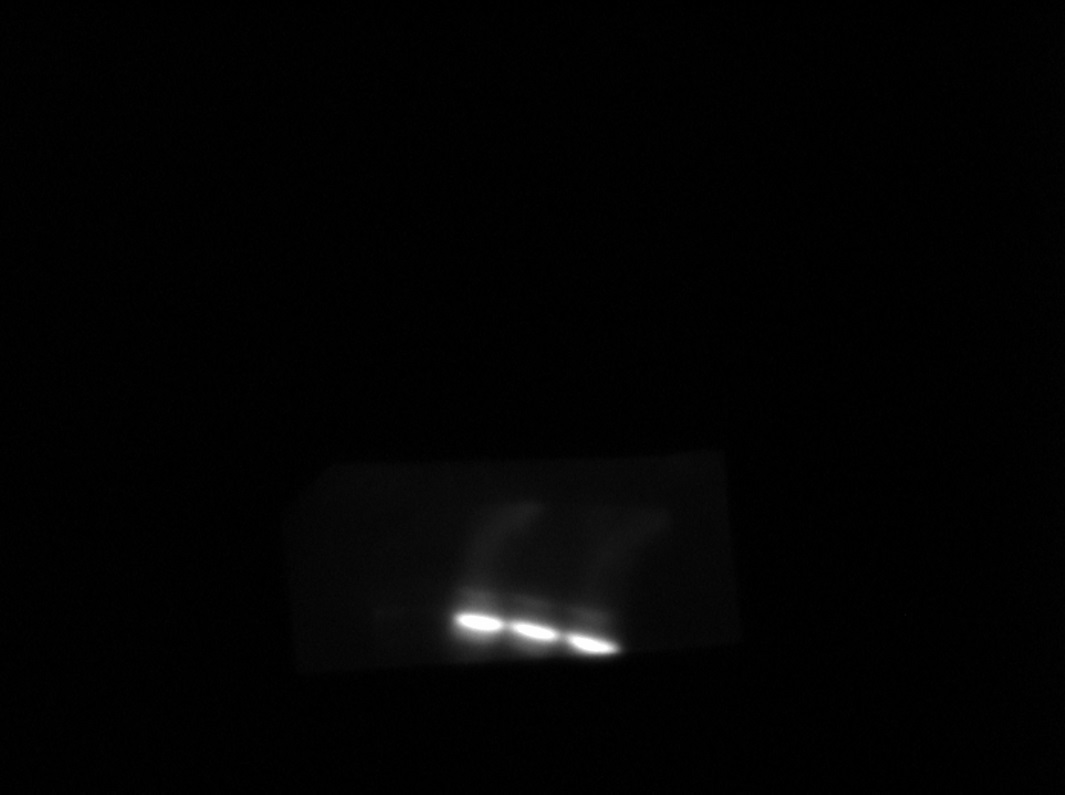

Supplement: Data S1 [file peerj-08-10148-s001.zip › raw data/Fig1/Fig1B GAPDH_siNC siSNIP1-330 siSNIP1-871.jpg]

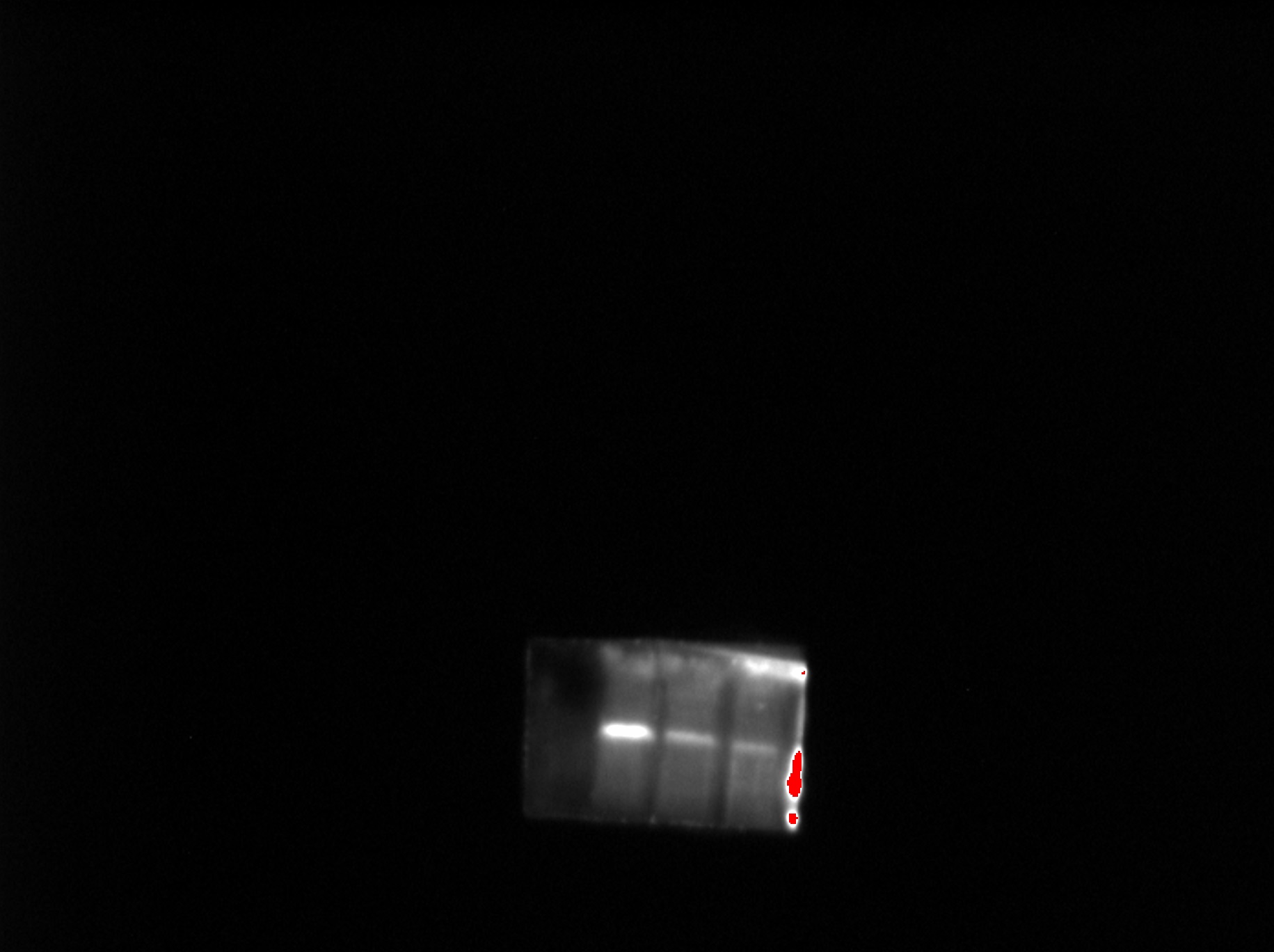

Supplement: Data S1 [file peerj-08-10148-s001.zip › raw data/Fig1/Fig1B wb snip1_siNC siSNIP1-330 siSNIP1-871.jpg]

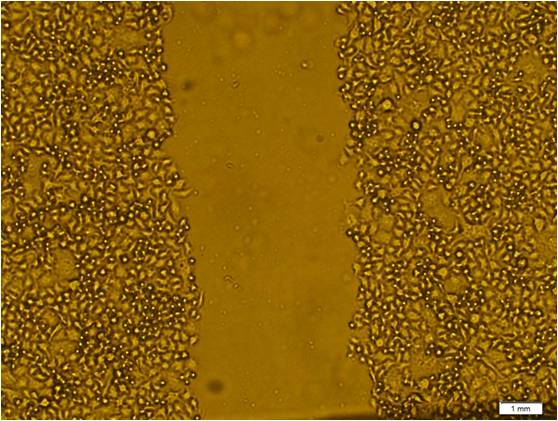

Supplement: Data S1 [file peerj-08-10148-s001.zip › raw data/Fig1/Fig1C siNC 0hr.jpg]

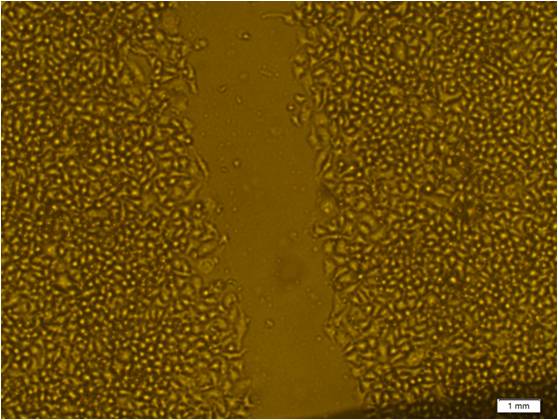

Supplement: Data S1 [file peerj-08-10148-s001.zip › raw data/Fig1/Fig1C siNC 12hr.jpg]

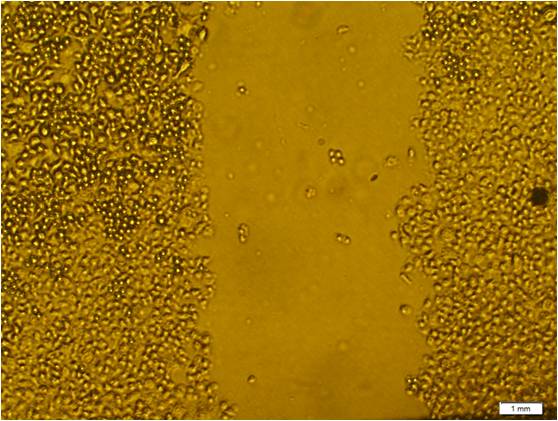

Supplement: Data S1 [file peerj-08-10148-s001.zip › raw data/Fig1/Fig1C siSNIP1-330 0hr.jpg]

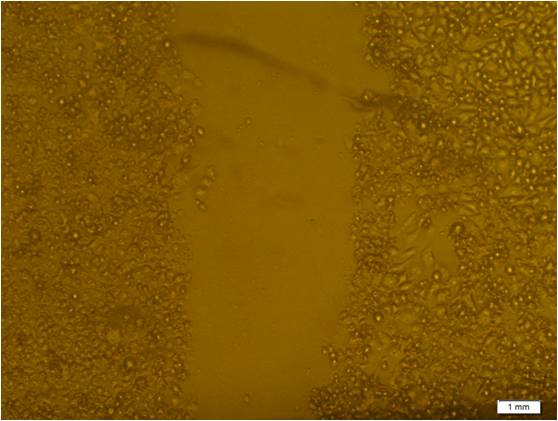

Supplement: Data S1 [file peerj-08-10148-s001.zip › raw data/Fig1/Fig1C siSNIP1-330 12hr.jpg]

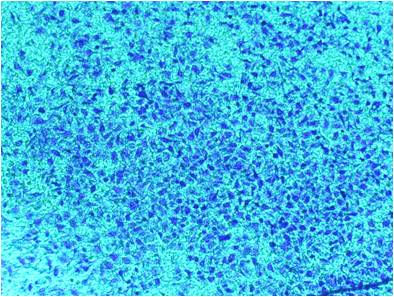

Supplement: Data S1 [file peerj-08-10148-s001.zip › raw data/Fig1/Fig1D siNC 24h.jpg]

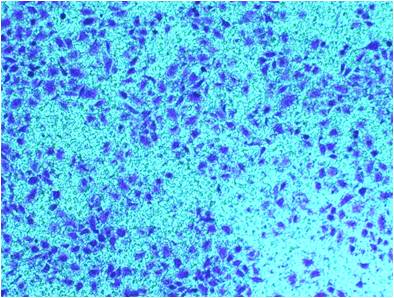

Supplement: Data S1 [file peerj-08-10148-s001.zip › raw data/Fig1/Fig1D siSNIP1-330 24h.jpg]

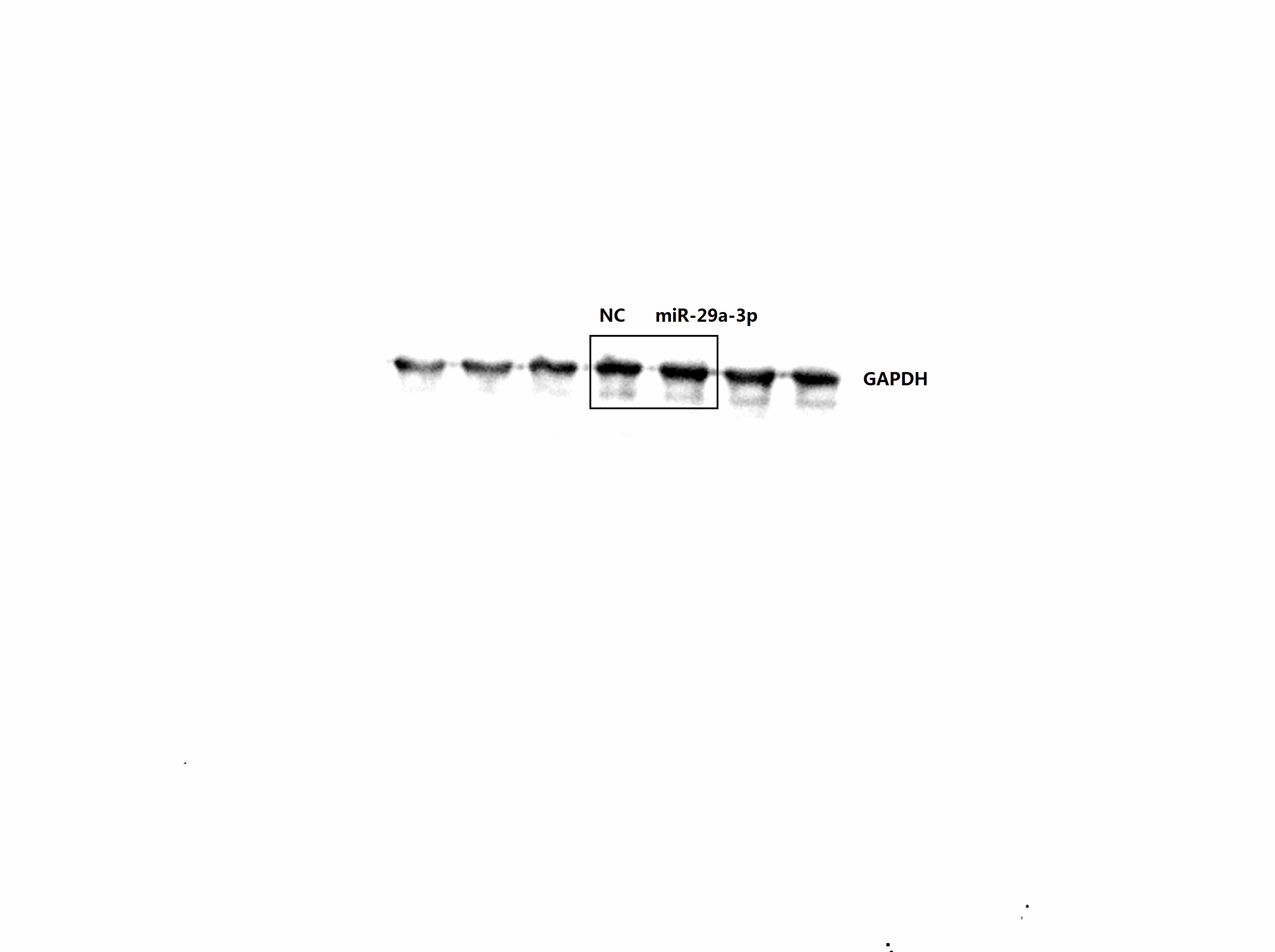

Supplement: Data S1 [file peerj-08-10148-s001.zip › raw data/Fig2/Fig2F GAPDH wb_NC miR-29a-3p.jpg]

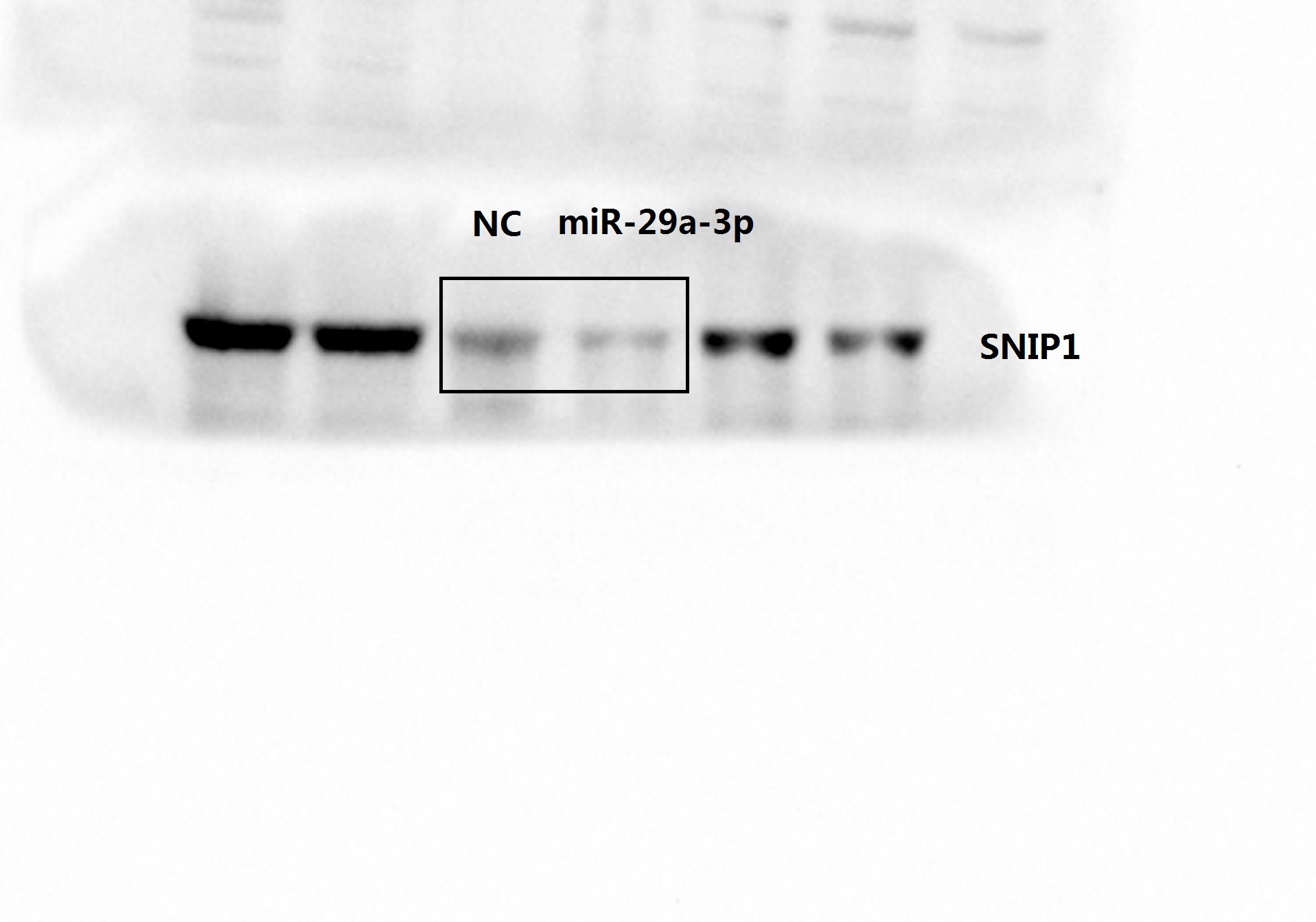

Supplement: Data S1 [file peerj-08-10148-s001.zip › raw data/Fig2/Fig2F SNIP1 wb_NC miR-29a-3p.jpg]

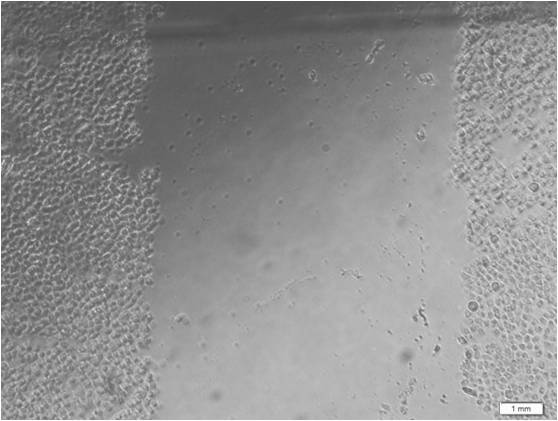

Supplement: Data S1 [file peerj-08-10148-s001.zip › raw data/Fig3/Fig3A miR-29a-3p 0 h.jpg]

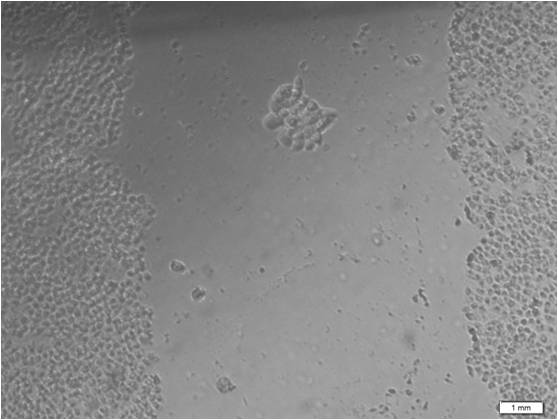

Supplement: Data S1 [file peerj-08-10148-s001.zip › raw data/Fig3/Fig3A miR-29a-3p 12 h.jpg]

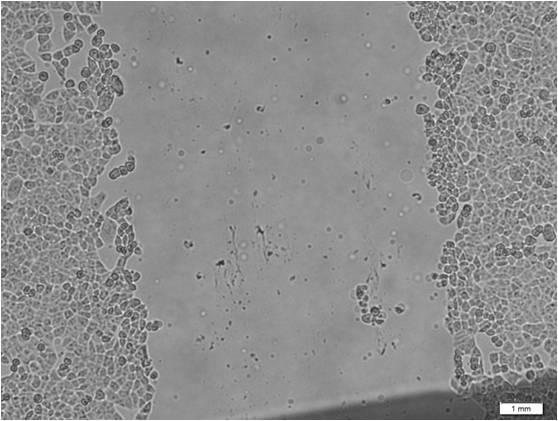

Supplement: Data S1 [file peerj-08-10148-s001.zip › raw data/Fig3/Fig3A NC 0 h.jpg]

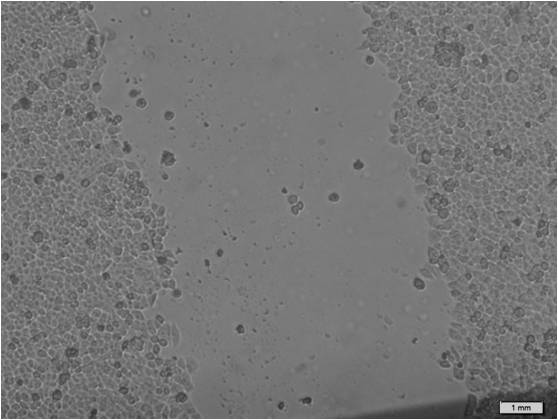

Supplement: Data S1 [file peerj-08-10148-s001.zip › raw data/Fig3/Fig3A NC 12 h.jpg]

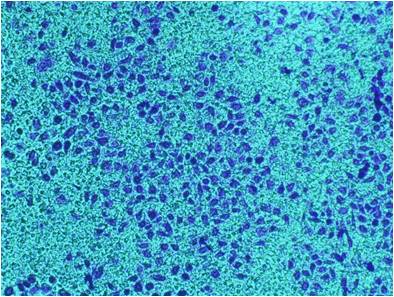

Supplement: Data S1 [file peerj-08-10148-s001.zip › raw data/Fig3/Fig3C miR-29a-3p.jpg]

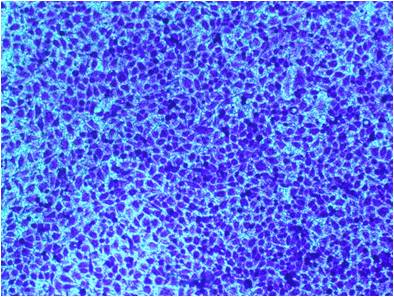

Supplement: Data S1 [file peerj-08-10148-s001.zip › raw data/Fig3/Fig3C NC.jpg]

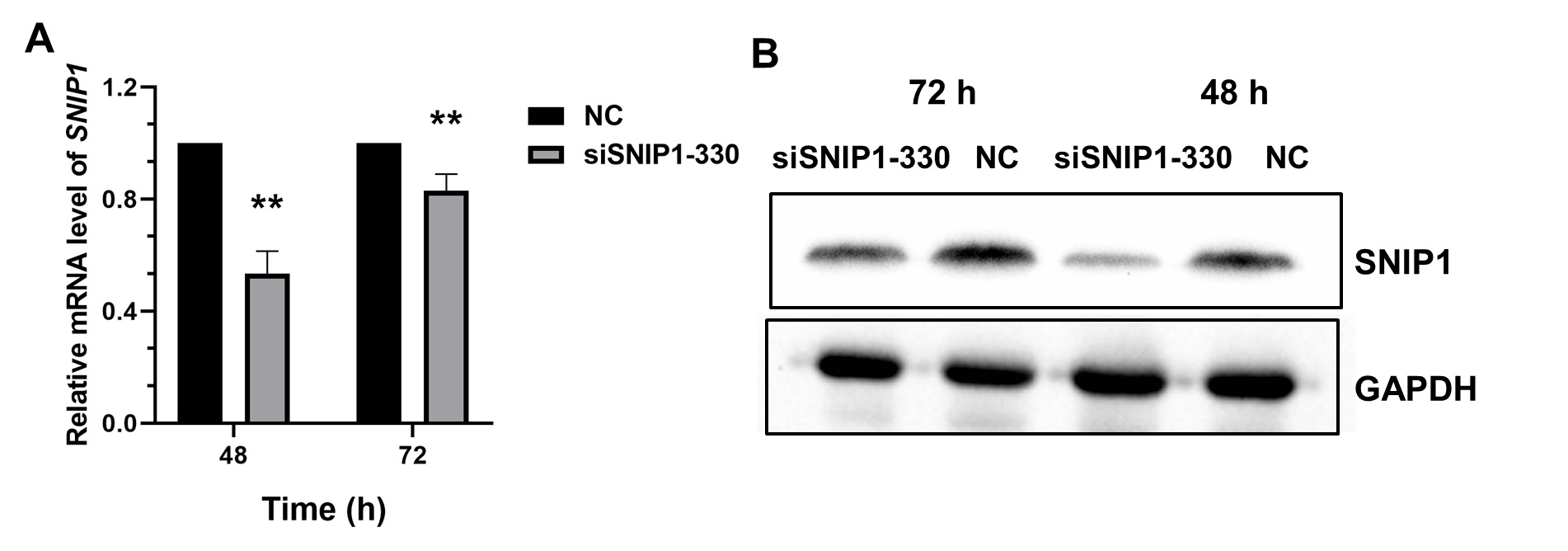

Supplement: Figure S1 — (A-B) RT-qPCR and Western blot analysis of SNIP1 expression in HeLa cells after transfected with siSNIP1-330 or siNC at 48 h and 72 h, respectively. [file peerj-08-10148-s002.jpg]

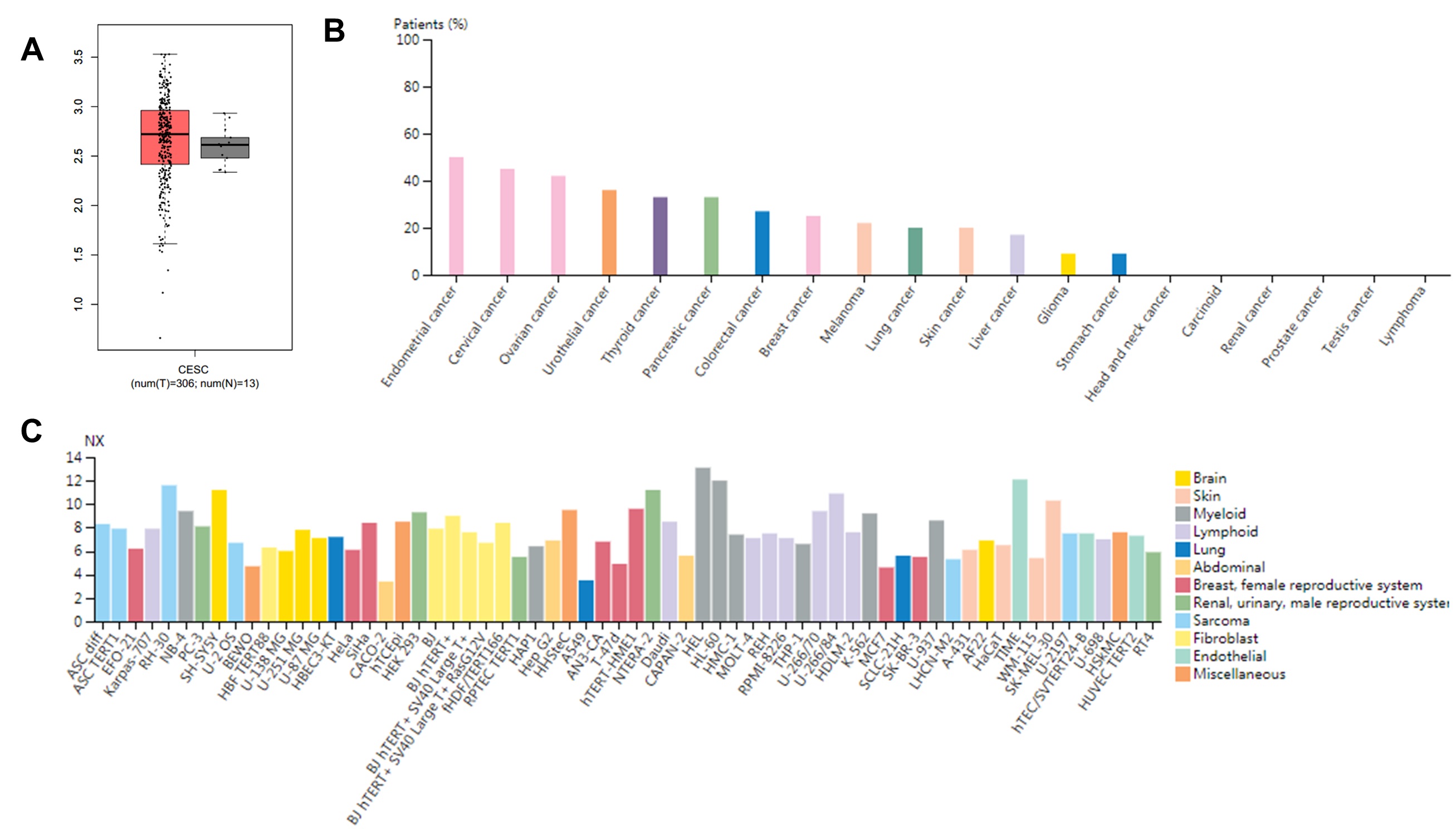

Supplement: Figure S2 — (A) Differential expression levels of SNIP1 from cervical cancer samples vs normal samples analyzed from GEPIA (http://gepia.cancer-pku.cn/). (B) Protein expression of SNIP1 in different cancer patients analyzed from HPA (https://www.proteinatlas.org/). 5/11 patients with cervical cancer show medium expression. (C) RNA expression of SNIP1 in different cell lines analyzed from HPA (https://www.proteinatlas.org/). [file peerj-08-10148-s003.jpg]
